# Prognostic value of C-reactive protein-to-albumin ratio in acute pancreatitis: a systematic review and meta-analysis

**DOI:** 10.12688/f1000research.134938.2

**Published:** 2023-09-22

**Authors:** I Ketut Mariadi, Gde Somayana, Christina Permata Shalim, Dwijo Anargha Sindhughosa, Dian Daniella, Made Lady Adelaida Purwanta

**Affiliations:** 1 Division of Gastroenterology and Hepatology, Faculty of Medicine, Udayana University/Prof Dr. I.G.N.G. Ngoerah Hospital, Denpasar, Bali, Indonesia; 2 Siloam Hospitals Kebon Jeruk, West Jakarta, Jakarta, Indonesia; 3 Department of Internal Medicine, Faculty of Medicine, Udayana University/Prof Dr. I.G.N.G. Ngoerah Hospital, Denpasar, Bali, Indonesia; 4 Intern Doctor, Internal Medicine Department, Bali Mandara Hospital, Denpasar, Bali, Indonesia

**Keywords:** c-reactive protein, albumin, acute pancreatitis, prognostic, meta-analysis

## Abstract

**Background**: Acute pancreatitis (AP) is a common disorder and although most of the cases are mild, the mortality risk is high when it comes to severe AP. It is therefore important to determine the severity of AP as early as possible. This review aimed to determine the prognostic value of C-reactive protein-to-albumin ratio (CRP/alb ratio) in patients with AP.

**Methods**: We performed a systematic search on the electronic databases PubMed, Science Direct, and Cochrane Library up to January 2023. Studies reporting CRP/alb ratio on admission and its association with severity or mortality in AP patients were included. We calculated pooled mean difference (MD) and their 95% confidence intervals (CI) using a random-effects model. Quality assessment of the included studies was appraised using a Newcastle–Ottawa scale.

**Results**: A total of six studies comprising 2244 patients were included in this meta-analysis. Severe AP had higher CRP/alb ratio on admission than mild-moderate AP (pooled MD: 3.59; 95% CI: 2.51-4.68; p<0.00001). CRP/alb ratio was also significantly higher on non-survivor AP patients compared to survivor AP patients (pooled MD: 2.12; 95% CI: 0.43-3.8; p < 0.01).

**Conclusion**: High CRP/alb ratio can be used as an early predictor of poor prognosis in patients with AP.

## Introduction

Acute pancreatitis (AP) is an inflammation of the pancreas characterized by sudden and severe onset of abdominal pain and elevated pancreatic enzyme. ^1^^,^^2^ This condition is mostly caused by bile stones or heavy use of alcohol. Even though most of the cases are mild, the mortality risk is high when it comes to severe AP. AP is a common gastrointestinal disease with reported global incidence rate of 34.8 per 100,000 population in 2019. ^3^ The overall mortality rate of AP is 3% to 10%, but in severe AP the mortality rate rises to 36% to 50%. ^4^ Thus, early determination of the disease severity to choose appropriate therapeutic strategy are of great importance.

Several scoring systems are commonly used to determine the severity and prognosis of AP, such as Ranson scores, bedside index for severity in acute pancreatitis (BISAP), acute physiological assessment and chronic health evaluation II (APACHE II), and Atlanta classification. ^5^ These scoring systems use multiple blood test results and clinical parameters, and almost all of them need repeated blood tests 48 hours after admission to increase its accuracy on predicting AP severity. There is a need for tools to predict the prognosis of AP within the first hour of admission.

C-reactive protein (CRP) is an inflammatory marker that is widely used in clinical practice to determine the severity of various inflammatory and infective conditions. Inflammation and infection boost this liver-produced acute phase reactant. ^6^ Albumin, on the other hand, is a liver-produced negative acute phase reactant that diminishes during inflammation. Albumin is also associated with disease severity and mortality. ^7^

CRP-to-albumin (CRP/alb) ratio was recently discovered as a new prognostic score associated with inflammation severity and mortality even though there is still no consensus on normal values of CRP/alb ratio. Some studies already tried to establish the relationship between CRP/alb ratio to AP severity and mortality and most of them yielded positive results. However, there is no meta-analysis that is currently available that summarizes all of these studies findings.

In this study, a systematic review and meta-analysis were conducted to investigate the prognostic value of CRP/alb ratio in AP.

## Methods

This systematic review and meta-analysis conforms to the Preferred Reporting Items for Systematic Reviews and Meta-Analyses (PRISMA) standards. We registered this review in PROSPERO (registration number: CRD42023427438).

### Literature search

We performed a systematic literature search using PubMed, Science Direct, and Cochrane Library to find eligible journals from their commencement to January 31st, 2023. We employed keywords “pancreatitis” AND (“CRP albumin ratio” OR “C-reactive protein/albumin” OR “C-reactive protein albumin” OR “CRP/albumin” OR “CRP alb ratio” OR “CRP/alb”). Additionally, we examine the references of pertinent articles. Duplicate results were removed after the initial search.

### Study selection

Three authors (IKM, CPS, DAS) independently performed study selection. A screening of study titles and abstracts was undertaken to exclude irrelevant literature. The inclusion and exclusion criteria for this review were applied to studies that passed the initial screening. The studies were included if they met all of the mentioned criteria: (1) observational studies reporting patients with acute pancreatitis, (2) reporting CRP/alb ratio on admission, (3) adult patients, (4) reporting the AP severity and/or patient’s mortality, (5) articles in English or Indonesian. Moreover, the studies were excluded if they meet one of the following criteria: (1) no full text available, (2) case reports, (3) conference papers, (4) review articles, (5) non research letters and (6) commentaries, (7) did not provide the necessary data for conducting meta-analysis.

### Data extraction

Data of the included studies that were extracted are the first author’s name, year of publication, country, type of study, number of patients, age, CRP/alb ratio means or median value, and outcomes (severity or mortality).

The primary outcome studied in the present systematic review and meta-analysis is severity of AP. The secondary outcome was the mortality of AP patients. All authors utilized an electronic data collection form to acquire the necessary information from each article.

### Risk of bias

The Newcastle–Ottawa scale (NOS) was adopted to assess the risk of bias in each study included. Three authors (IKM, CPS, DAS) independently conducted this process. The assessment were divided into three categories: low risk (7-9), moderate risk (4–6), and high risk (0-3).

### Statistical analysis

Review Manager 5.4 and Stata 17 were used as the softwares for statistical analysis. We estimated the pooled mean difference (MD) with 95% confidence intervals (CI) using the mean difference (MD) and standard deviation (SD) from each study. We utilized a calculator by Luo et al. and Wan et al. to determine the mean if data were provided as median with Q1 and Q3 or range. ^8^^,^^9^ Heterogeneity was assessed using the I ^2^ statistic, which reveals which percentage of the variation in observed impacts across studies is related to the variation in true effects, with values greater than 60% indicating significant heterogeneity. All P values were two-tailed, and <0.05 was regarded statistically significant. Forest plot was generated to give a visual suggestion of the amount of study heterogeneity and the estimated effect. The leave-one-out method (repeating the analysis after eliminating one study at a time) was used to conduct a sensitivity analysis.

## Results

### Study selection and characteristics

**Figure 1.**
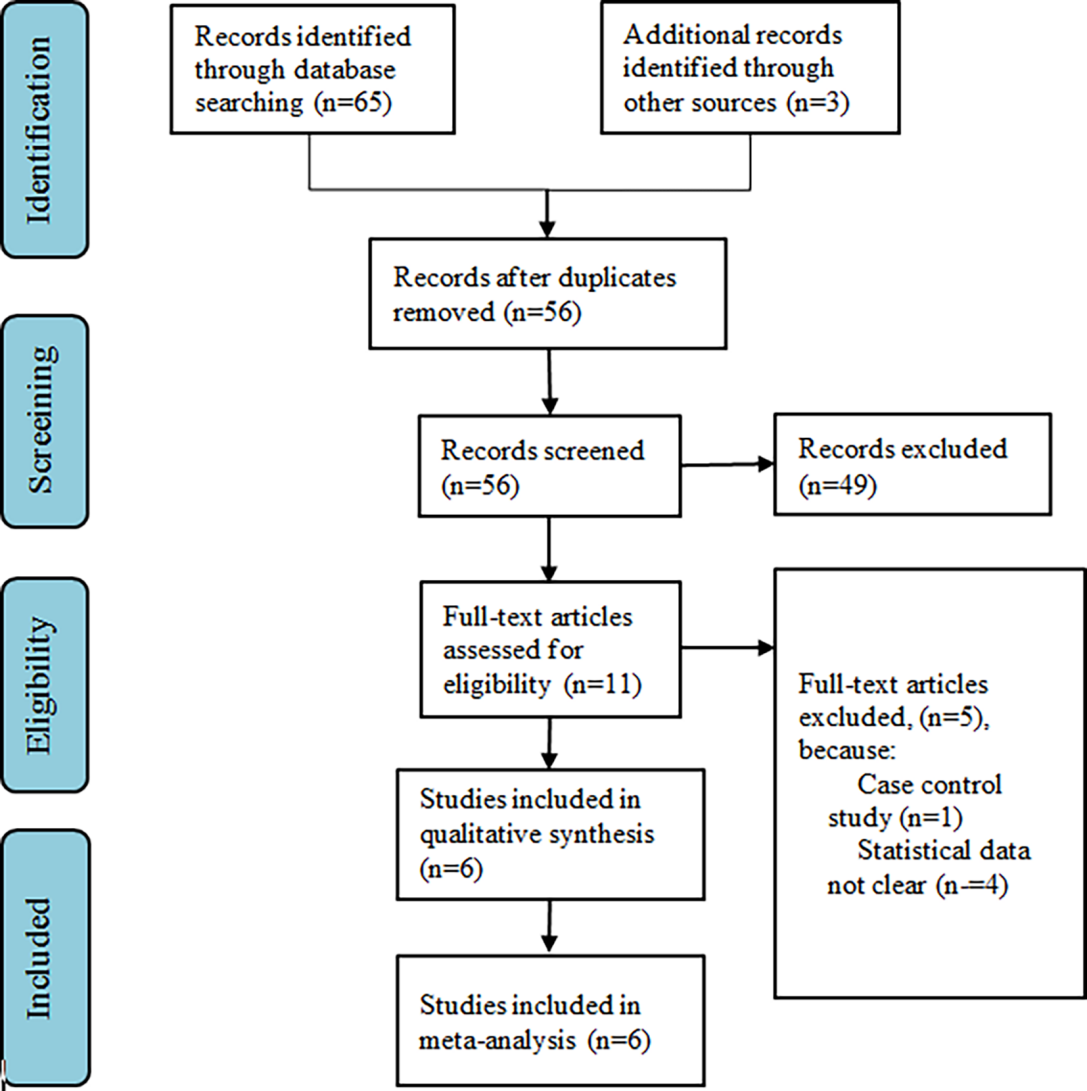
PRISMA flow diagram.

The keywords search yielded a total of 21 publications. After eliminating the duplicates, we retrieved 18 publications. By screening the titles and abstracts, we excluded 7 studies, leaving us with 11 potential studies. Then, the full texts of the potential studies were obtained and reviewed to see if they were eligible for inclusion in the meta-analysis. Publications that did not offer all the necessary data for this meta-analysis and that did not fulfill all the inclusion criteria were excluded. Thereby, in the present study, a total of 6 studies were included. ^10^^–^^15^ PRISMA study flow diagram is described in Figure 1.

All 6 included studies were retrospective cohorts, with a total of 2244 patients. All studies were published in 2017–2022. Based on the study location, 4 studies were conducted in Turkey and 2 in China. There were 5 studies that evaluated CRP/alb relationship with AP severity. Meanwhile, the relationship between CRP/alb ratio with AP mortality were evaluated in 2 studies. Table 1 showed the characteristics of the included studies.

**Table 1.** Characteristics of the studies included in the meta-analysis.

Study	Country	Study design	Assessment of severity	Sample	Mean/median age	Non-severe/severe	Survivors/non-survivors	Cut-off severe	Cut-off mortality
Karabuga 2022	Turkey	retrospective observational study	BISAP score	female 253 (50.6%) male 247 (49.4%)	55.68 ± 18.30	mild 388 severe 112	survivor 473 death 23	>0.0015	
Kiyak 2022	Turkey	retrospective observational study	Balthazar and Ranson score	female 173 (52.6%) male 156 (47.5%)	male 50.3 ± 15.7 female 54.3 ± 17.4	mild 238 severe 91		>5.34	Mortality (-) 3.3 ± 2.6 (+) 6.5 ± 1.2
Ugurlu 2022	Turkey	retrospective observational study	contrast-enhanced abdominal computed tomography (CECT) and Revised Atlanta Classification	582 female 344 (59.1%) male 238 (40.8%)	male 58.06 ± 17.34 female 57.9 ± 21.05	AEP 525 ANP 57	survivor 541 death 41	> 0.878	
Yilmaz 2018	Turkey	retrospective observational study	Ranson score	264 female 159 (60.2%) male 105 (39.8%)	59.97 ± 17.47	moderate 204 severe 60	no mortality	>8.51	
Zhao 2020	China	retrospective observational study		140 female 42 (30%) male 98 (70%)	49.88 ± 13.94		survivor 124 death 16		>7.69
Zhao 2023	China	retrospective observational study	severe AP defined as persistent single or multiple organ failure (>48 h)	284	59.50 (IQR 39.00–70.00)	non severe 249 severe 35	survivor 273 death 11	>5.03	>5.33

### Quality assessment

Using the NOS to evaluate the risk of bias, five studies were found to be at low risk, while one study was at moderate risk. Table 2 showed the risk of bias assessment.

**Table 2.** NOS of the studies included in the meta-analysis.

Study	Selection				Comparability	Outcome				
	Representativeness of the exposed cohort	Selection of the nonexposed cohort	Ascertainment of exposure	Demonstration that outcome of interest was not present at start of study	Comparability of Cohorts	Assessment of outcome	Was follow-up long enough for outcomes to occur	Adequacy of follow up of cohorts	SCORE	Evidence quality
Karabuga 2022	*	*	*		*	*	*	*	7	Low risk of bias
Kiyak 2022	*	*	*			*	*	*	6	Low risk of bias
Ugurlu 2022	*	*	*	*	*	*	*	*	8	Low risk of bias
Yilmaz 2018		*		*		*	*	*	5	Moderate risk of bias
Zhao 2020	*	*	*	*		*	*	*	7	Low risk of bias
Zhao 2023	*	*	*		*	*	*	*	7	Low risk of bias

### CRP/alb ratio and AP severity

**Figure 2.**
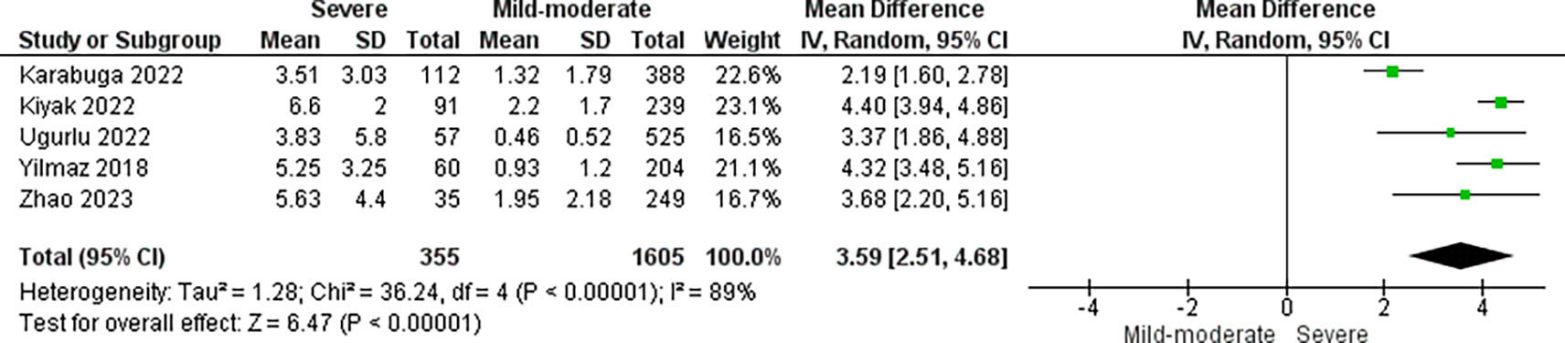
Forest plot of studies comparing CRP/alb ratio in severe vs mild-moderate AP patients.

**Figure 3.**
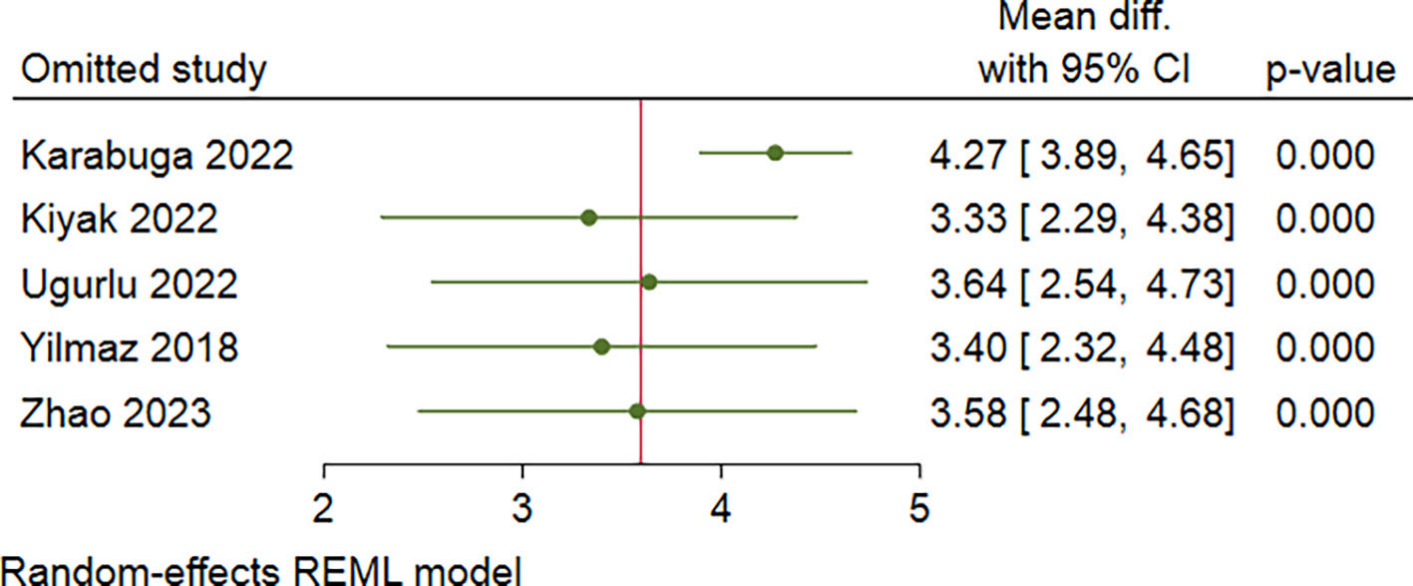
Leave-one-out analysis of CRP/alb ratio comparison of severe and mild-moderate AP patients.

Five studies with a total of 1960 patients analyzed the relationship between CRP/alb ratio and AP severity. Severe AP patients had higher CRP/alb ratio than mild-moderate AP patients (pooled MD: 3.59; 95% CI: 2.51-4.68; p < 0.00001) ( Figure 2). In addition, a sensitivity analysis was performed with leave-one-out method due to severe heterogeneity (I ^2^ = 89%). Leave-one-out analysis showed no significant change in results after excluding one study at a time ( Figure 3).

### CRP/alb ratio and AP mortality

**Figure 4.**
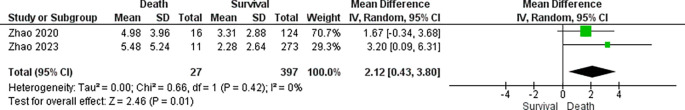
Forest plot of studies comparing CRP/alb ratio in survivor vs non survivor AP patients.

We found only two studies evaluating AP mortality and CRP/alb ratio that fulfilled the inclusion criteria. Heterogeneity in these studies are considered low with I2 = 0%, thus we used fixed-effect models in this analysis. Based on these analysis, non-survivor patients had higher CRP/alb ratio than survivor patients (pooled MD: 2.12; 95% CI: 0.43-3.8; p < 0.01) ( Figure 4).

## Discussion

In patients with AP there is a local inflammation that can cause systemic effects. This systemic inflammation is prone to develop systemic organ dysfunction and later organ failure. ^16^ Severity of AP is classified based on the presence of these systemic complications and organ failure, with severe AP is defined as persistent organ failure. Meanwhile in mild AP there are no systemic complications or organ failure. ^17^

Because the severity of AP is determined by how much inflammation there is, markers of inflammation are thought to determine the prognosis of AP. CRP, as one of the most commonly used biomarkers, has been shown to correlate well with AP severity. The problem was CRP levels alone at admission showed poor predictive value, and the correlation only significant when assessed later at 48 hours from admission. The accepted number by international consensus for prediction of severe AP was CRP >150 mg/L within the first 48 hours. ^18^ Several studies discovered that both CRP and albumin can be beneficial in prognosis determination of various diseases. Combination of these two markers is expected to produce a more superior predictive value than using only one of them.

In this meta-analysis, we noted that the CRP/alb ratio is a promising prognostic score that can be used for the prediction of severity and mortality in AP patients. We analyzed 6 studies that fulfilled our inclusion criteria. Four studies from Turkey and 2 studies from China. Several included studies have different measurement units of CRP and albumin and some did not mention specifically what measurement units that were used. We tried to contact each corresponding author of the study in question to clarify this problem but none gave a response, thus we estimate the measurement units based on other relevant data from each study and make it all in the same measurement units. Some studies actually passed the screening process and fulfilled the inclusion criteria but eventually excluded because the statistical data provided by those studies were not clear.

We found that severe and non-survivor AP patients had higher CRP/alb ratio at admission than those with non severe and survivor AP patients. These findings were in line with results from other studies in recent years that showed the benefit of CRP/alb ratio as a prognostic marker in a variety of diseases. Because inflammation and carcinogenesis are linked, several systematic studies revealed the predictive practicality of CRP/alb ratio in different types of cancers. ^19^^,^^20^ Several studies tried to determine this association focusing on critically ill patients. Park et al. in their single center retrospective study found out that higher CRP/alb ratio was associated with increased mortality in ICU patients. ^21^ Wang et al. discovered that critically ill acute kidney injury patients with greater CRP/alb ratios had higher in-hospital mortality and 2-year all-cause mortality. ^22^

The results from this current meta-analysis reveal that CRP/Alb ratio may also serve as a reliable prognostic marker in patients with AP. AP is a disease that in severe form often leads to admission in the intensive care unit. This might explain the similar findings of this current study and previous study that focuses on critically ill patients.

Based on our knowledge, this is the first meta-analysis that evaluates CRP/alb ratio as a prognostic marker in AP patients. There is one systematic review by Tarar et al. that also assessed the prognostic value of CRP/alb ratio in AP patients, but that study just did the qualitative analysis based on 3 retrospective cohort studies and did not conduct meta-analysis. That study discovered an overall beneficial connection between the CRP/alb ratio at admission and the occurrence of severe AP as well as a longer hospital stay. ^23^ These outcomes are consistent with the results of our study.

Nevertheless, our research has its own limitations. First, 6 studies that included in this meta-analysis only came from 2 countries, which are Turkey and China. We cannot explain this lack of variety in study location, but we can say that this lack of diversity has been a limitation in this current study. Second, the differences in the units of measurement that were used in each study lead to the possibility of inaccurate comparisons made in this study.

Lastly, almost every studies used different approach and criteria to differentiate severe AP with non severe AP. This difference were expected because each study was conducted in different countries and different centers. Each center usually has severity criteria of acute pancreatitis that is more often used than the other. But, we thought this difference did not significantly affect the results, ^24^^,^^25^ because all the criteria were standardized criteria that are often used and in few studies already compared and showed no significant differences in their prognostic value.

## Conclusion

Our meta-analysis showed that high CRP/Alb ratio is associated with severe AP and mortality in patients with AP. Thus, CRP/alb ratio can be used as an early predictor of poor prognosis in patients with AP. However, due to the study’s limitations, large-scale trials involving patients of various ethnicities will be needed to verify the results we obtained.

## Data Availability

No data are associated with this article. Zenodo: Prognostic value of C-reactive protein-to-albumin ratio in acute pancreatitis: a systematic review and meta-analysis (extended data), https://doi.org/10.5281/zenodo.8003368. ^26^ This project contains the following extended data:
- Search strategy.docx Zenodo: PRISMA checklist for ‘Prognostic value of C-reactive protein-to-albumin ratio in acute pancreatitis: a systematic review and meta-analysis (extended data)’, https://doi.org/10.5281/zenodo.8003368. ^26^ Data are available under the terms of the Creative Commons Attribution 4.0 International license (CC-BY 4.0).
