# Supplementary material for: Prognostic value of C-reactive protein-to-albumin ratio in acute pancreatitis: a systematic review and meta-analysis
Source: F1000Res. 2023 Sep 22;12:748. Originally published 2023 Jun 26. [Version 2] doi: 10.12688/f1000research.134938.2 (PMC10558981; doi:10.12688/f1000research.134938.2)
Supplement: Peer review history

# Reviewer report: Reviewer response for version 2

**DOI:** 10.5256/f1000research.156179.r208619

**Published:** 2023-10-06

**Authors:** Hasan Maulahela

**Affiliations:** 1 Division of Gastroenterology, Department of Internal Medicine, Faculty of Medicine, Cipto Mangunkusumo National General Hospital, University of Indonesia, Depok, West Java, Indonesia

The author has revised it according to the review, no further comments

Are the rationale for, and objectives of, the Systematic Review clearly stated?

Yes

Is the statistical analysis and its interpretation appropriate?

Yes

Are sufficient details of the methods and analysis provided to allow replication by others?

Partly

Are the conclusions drawn adequately supported by the results presented in the review?

Yes

Reviewer Expertise:

Gastroenterology

I confirm that I have read this submission and believe that I have an appropriate level of expertise to confirm that it is of an acceptable scientific standard.

# Reviewer report: Reviewer response for version 2

**DOI:** 10.5256/f1000research.156179.r208618

**Published:** 2023-10-03

**Authors:** Bogi Pratomo Wibowo

**Affiliations:** 1 Faculty of Medicine, Brawijaya University, Malang, East Java, Indonesia

I have already read the recent revisions and I think it is appropriate. The revision are sufficient and I can Approved them.

Are the rationale for, and objectives of, the Systematic Review clearly stated?

Yes

Is the statistical analysis and its interpretation appropriate?

Partly

Are sufficient details of the methods and analysis provided to allow replication by others?

Yes

Are the conclusions drawn adequately supported by the results presented in the review?

Yes

Reviewer Expertise:

NA

I confirm that I have read this submission and believe that I have an appropriate level of expertise to confirm that it is of an acceptable scientific standard.

# Reviewer report: Reviewer response for version 1

**DOI:** 10.5256/f1000research.148033.r182364

**Published:** 2023-07-26

**Authors:** Bogi Pratomo Wibowo

**Affiliations:** 1 Faculty of Medicine, Brawijaya University, Malang, East Java, Indonesia

**REVIEW ARTICLE**

 This article addresses the prognostic value of CRP/albumin ratio in severity and mortality of acute pancreatitis. It addresses an important topic, especially the high CRP/albumin ratio as an early predictor of severity to conduct the doctors in charge immediately choose an appropriate therapeutic strategy. Despite the rather small study included in this meta-analysis, it should make an important contribution.

 There are a number of issues with the inconsistency in the words chosen and the statistic analysis that need to be clarified. Below are more specific comments by section:

**Abstract**

 The title stated prognostic role, in my opinion it should be prognostic value in conjunction with the statement of prognostic value of C-reactive protein-to-albumin ratio on the background of the abstract. The conclusion also stated the poor prognostic seems to be uncorrelated with the data reported in the article. It would be better using the term of severity or mortality in state of poor prognostic.

**Introduction**

 More information about the epidemiology of acute pancreatitis would provide welcome context here. A bit more detail of incidence rate of acute pancreatitis in population would be helpful since you explicitly state acute pancreatitis is a common disorder on the background of the abstract.

**Results**

 On the page 6 of 9, the double subtitles of CRP/alb ratio and AP severity should not be used. In my opinion, the subtitle should be CRP/alb ratio and AP severity and CRP/alb ratio and AP mortality. It is based on the primary and secondary outcome studied in this meta-analysis.

 Table 2 – you indicate one study is moderate risk of bias with score 5, but in the text report of quality assessment is written high risk. This should be minimalized by cross-checking between the table shows and the text reported.

 Figure 2 and 3 – the titles of forest plot are severe vs non severe patients, since you already make a case definition of non-severe as mild-moderate patients. It would be consistent if you use the title of severe vs mild-moderate patients.

 Figure 4 – the heterogeneity of the forest plot is I ^2^=0% which means low heterogeneity, with p value 0.42 which means the heterogeneity test is not significant, so it would be better if you used the Fixed model rather than Random model analysis.

**Discussion**

 Discussion of the CRP/alb ratio in cancer and critically ill patients is interesting, but it could be made much more succinct in keeping with the focus of acute pancreatitis patients on this article. More detail of the cut-off level of CRP/alb ratio here would be welcome.

Are the rationale for, and objectives of, the Systematic Review clearly stated?

Yes

Is the statistical analysis and its interpretation appropriate?

Partly

Are sufficient details of the methods and analysis provided to allow replication by others?

Yes

Are the conclusions drawn adequately supported by the results presented in the review?

Yes

Reviewer Expertise:

Gastroenterologist

I confirm that I have read this submission and believe that I have an appropriate level of expertise to confirm that it is of an acceptable scientific standard, however I have significant reservations, as outlined above.

# Response

**Published:** 2023-09-16

**Authors:** I Ketut Mariadi

**Affiliations:** Universitas Udayana, Denpasar, Bali, Indonesia

Dear Dr. Bogi Pratomo Wibowo,

 We sincerely appreciate the time and consideration you have provided in reviewing our manuscript.

 Here we provide responses to your comments:

**Abstract**

 The title stated prognostic role, in my opinion it should be prognostic value in conjunction with the statement of prognostic value of C-reactive protein-to-albumin ratio on the background of the abstract. The conclusion also stated the poor prognostic seems to be uncorrelated with the data reported in the article. It would be better using the term of severity or mortality in state of poor prognostic.

**Our response**: Thank you for your suggestion. After the discussion by the authors, we agreed that “prognostic value” was more suitable for this study title, thus we changed the title with “Prognostic value of C-reactive protein-to-albumin ratio in acute pancreatitis: a systematic review and meta-analysis”.

 For the conclusion in the abstract we tried to summarize our findings. Our findings showed that high CRP/alb ratio is associated with severe AP and mortality in patients with AP, thus we conclude that findings as poor prognostic. In the conclusion section in the full text we describe it in more detail than in the abstract.

**Introduction**

 More information about the epidemiology of acute pancreatitis would provide welcome context here. A bit more detail of incidence rate of acute pancreatitis in population would be helpful since you explicitly state acute pancreatitis is a common disorder on the background of the abstract.

**Our response**: Thank you for your suggestion. We already added data of acute pancreatitis incidence rate in the introduction section as you suggested.

**Results**

 On the page 6 of 9, the double subtitles of CRP/alb ratio and AP severity should not be used. In my opinion, the subtitle should be CRP/alb ratio and AP severity and CRP/alb ratio and AP mortality. It is based on the primary and secondary outcome studied in this meta-analysis.

**Our response**: Thank you for the correction. We already corrected the subtitles to the correct one: “CRP/alb ratio and AP severity” and “CRP/alb ratio and AP mortality”.

 Table 2 – you indicate one study is moderate risk of bias with score 5, but in the text report of quality assessment is written high risk. This should be minimalized by cross-checking between the table shows and the text reported.

**Our response**: Thank you for the correction. We already corrected the mistakes. The score range of Newcastle-Ottawa Scale should be: low risk (7-9), moderate risk (4–6), and high risk (0-3). The one study that you mentioned has a score of 5, thus considered moderate risk of bias.

 Figure 2 and 3 – the titles of forest plot are severe vs non severe patients, since you already make a case definition of non-severe as mild-moderate patients. It would be consistent if you use the title of severe vs mild-moderate patients.

**Our response**: Thank you for your suggestion. We already changed the title of figure 2 and 3 with severe vs mild-moderate patients as you suggested.

 Figure 4 – the heterogeneity of the forest plot is I ^2^=0% which means low heterogeneity, with p value 0.42 which means the heterogeneity test is not significant, so it would be better if you used the Fixed model rather than Random model analysis.

**Our response**: Thank you for your suggestion. We already changed the statistical models that we used for the analysis between CRP/alb ratio with mortality as shown in figure 4 with the fixed-effect model as you suggested.

**Discussion**

 Discussion of the CRP/alb ratio in cancer and critically ill patients is interesting, but it could be made much more succinct in keeping with the focus of acute pancreatitis patients on this article. More detail of the cut-off level of CRP/alb ratio here would be welcome.

**Our response**: Thank you for your suggestion. We already reduced the discussion about CRP/alb ratio in critically ill patients to keep the discussion focus on CRP/alb ratio in acute pancreatitis patients.

 Thank you for your attention. 

 Sincerely,

 I Ketut Mariadi

 Division of Gastroenterology and Hepatology, Faculty of Medicine, Udayana University/Prof Dr. I.G.N.G. Ngoerah Hospital, Denpasar, Bali, Indonesia

# Reviewer report: Reviewer response for version 1

**DOI:** 10.5256/f1000research.148033.r182368

**Published:** 2023-07-24

**Authors:** Hasan Maulahela

**Affiliations:** 1 Division of Gastroenterology, Department of Internal Medicine, Faculty of Medicine, Cipto Mangunkusumo National General Hospital, University of Indonesia, Depok, West Java, Indonesia

This is an important topic in the search for a simple prognostic marker for acute pancreatitis. The method chosen by the authors is strong (meta-analysis). Some additional information should be stated in the manuscript:

1. In the methods, who performed the literature search and what are their qualifications?
2. Any other attempts to acquire unpublished data?
3. From the studies we see that the severity criteria used are different for each study, how do the authors analyze this? Please explain more about it.
4. Explain more the possibility of more robust studies being excluded from the analysis because the authors cannot obtain the data (in the discussion).
5. Is there any risk of bias concerning the patient characteristics from the studies that were included in the analysis?

Are the rationale for, and objectives of, the Systematic Review clearly stated?

Yes

Is the statistical analysis and its interpretation appropriate?

Yes

Are sufficient details of the methods and analysis provided to allow replication by others?

Partly

Are the conclusions drawn adequately supported by the results presented in the review?

Yes

Reviewer Expertise:

Gastroenterology

I confirm that I have read this submission and believe that I have an appropriate level of expertise to confirm that it is of an acceptable scientific standard, however I have significant reservations, as outlined above.

# Response

**Published:** 2023-09-16

**Authors:** I Ketut Mariadi

**Affiliations:** Universitas Udayana, Denpasar, Bali, Indonesia

Dear Dr. Hasan Maulahela,

 We sincerely appreciate the time and consideration you have provided in reviewing our manuscript.

 Here we provide responses to your comments:

 1. In the methods, who performed the literature search and what are their qualifications?

 Our response: The literature search and study selection performed by three authors (IKM, CPS, DAS) independently. IKM and DAS are internists in the Division of Gastroenterology and Hepatology, Faculty of Medicine, Udayana University/Prof Dr. I.G.N.G. Ngoerah Hospital, Denpasar, Bali, Indonesia. While CPS is a medical doctor at one of hospital in Jakarta

 2. Any other attempts to acquire unpublished data?

 Our response: We are not seeking any unpublished data since we could not determine the quality of the study. However, we try to obtain any incomplete data by tracking the supplementary information of the respective articles in our study.

 3. From the studies we see that the severity criteria used are different for each study, how do the authors analyze this? Please explain more about it.

 Our response: Thank you for your suggestion. We have added the discussion about different severity criteria that were used in each study in the “Discussion” section. Different severity criteria from each study were expected because each study was conducted in different countries and different centers. Each center usually has severity criteria of acute pancreatitis that is more often used than the other. But, we thought this difference did not significantly affect the results, because all the criteria were standardized criteria that are often used and in few studies already compared and showed no significant differences in their prognostic value.

 4. Explain more the possibility of more robust studies being excluded from the analysis because the authors cannot obtain the data (in the discussion).

 Our response: Thank you for your suggestion. We added the discussion about this in the discussion as you suggested. Some studies passed the screening process and fulfilled the inclusion criteria but eventually excluded because the statistical data provided by those studies were not clear. We tried to contact the corresponding author for those studies but none gave us response, thus the studies were excluded.

 5. Is there any risk of bias concerning the patient characteristics from the studies that were included in the analysis?

 Our response: Based on the  risk of bias that we conducted using Newcastle-Ottawa Scale, most of the studies included were considered low risk of bias, with the exposed and non exposed individuals considered truly or somewhat representative of the target population.

 Thank you for your attention. 

 Sincerely,

 I Ketut Mariadi

 Division of Gastroenterology and Hepatology, Faculty of Medicine, Udayana University/Prof Dr. I.G.N.G. Ngoerah Hospital, Denpasar, Bali, Indonesia
